# Towards Better Health, Social, and Community-Based Services Integration for Patients with Chronic Conditions and Complex Care Needs: Stakeholders’ Recommendations

**DOI:** 10.3390/ijerph17228437

**Published:** 2020-11-14

**Authors:** Catherine Hudon, Maud-Christine Chouinard, Marie-Dominique Beaulieu, Mathieu Bisson, Danielle Bouliane, Martine Couture, Serge Dumont, Antoine Groulx, Véronique Sabourin

**Affiliations:** 1 Department of Family Medicine and Emergency Medicine, University of Sherbrooke, Sherbrooke, QC J1K 2R1, Canada; Mathieu.Bisson2@usherbrooke.ca; 2 CHUS Research Center, Sherbrooke, QC J1H 5N4, Canada; vero.sabourin75@gmail.com; 3 Faculty of Nursing, Montreal University, Montréal, QC H3T 1A8, Canada; maud.christine.chouinard@umontreal.ca; 4 Department of Family Medicine and Emergency Medicine, Montreal University, Montréal, QC H3T 1J4, Canada; marie-dominique.beaulieu@umontreal.ca; 5 Saguenay-Lac-Saint-Jean Integrated University Health and Social Services Center, Chicoutimi, QC G7H 7K9, Canada; dbouliane1@hotmail.com (D.B.); martinecouture2017@gmail.com (M.C.); 6 Faculty of Social Sciences, School of Social Work and Criminology, Laval University, Québec, QC G1V 0A6, Canada; serge.dumont.1@ulaval.ca; 7 Alliance santé Québec, Québec, QC G1V 0A6, Canada; antoine.groulx@fmed.ulaval.ca

**Keywords:** integrated services, primary care, complex health needs, patient-oriented research

## Abstract

The objective was to report on issues related to patients with complex care needs and recommendations identified by 160 key participants at a summit in Quebec City about better integration of primary health care services for patients with chronic diseases and complex care needs. A descriptive qualitative approach was used. While focus groups were led by a facilitator, a rapporteur noted highlights and a research team member took independent notes. All notes were analyzed by using a thematic analysis according to an inductive method. Seven issues were identified, leading to the formulation of recommendations: (1) valuing the experience of the patient; (2) early detecting of a non-homogeneous patient population; (3) defining interprofessional collaboration based on patient needs; (4) conciliating services provided by clinical settings according to a registered clientele-based logic with the population-based logic; (5) working with the community sector; (6) aligning patient-oriented research values with existing challenges to primary care integration; and (7) promoting resource allocation consistent with targeted recommendations. The summit highlighted the importance of engaging all stakeholders in improvement of integrated care for patients with complex care needs. The resulting recommendations target shared priorities towards better health, social, and community-based services integration for these patients.

## 1. Introduction

In Canada, as in many other industrialized countries [1,2], 10% of the population consumes 80% of health care and psychosocial services [3]. Due to the interaction of multiple chronic conditions with mental health disorders and/or socioeconomic vulnerability, these people may have more complex care needs that must be taken into account when allocating resources [4,5]. For this reason, interprofessional collaboration and integration between health services and social services are essential [4,5,6]. However, health care and psychosocial services, including community-based organizations, are often provided in silos, resulting in significant barriers to information flow and to the harmonization of follow ups between practitioners [7,8,9].

Primary care represents the services integration cornerstone of a health care system [10,11,12,13]. In Quebec, the majority of these services are delivered through a Family Medicine Group (FMG), which is comprised of an interprofessional team that is responsible for a registered clientele. Emergency, psychosocial and home care services are provided through Integrated Health and Social Services Centres (IHSSC) and Integrated University Health and Social Services Centres (IUHSSC) that are responsible for the population of a designated territory [14].

In a context where we must contemplate solutions to meet the challenges encountered in following up with these patients [4,15,16,17], a summit gathering a critical mass of key stakeholders was organized as part of the Quebec Strategy for Patient-Oriented Research (SPOR) Support Unit for Public and Patient-Oriented Research and Trials (SUPPORT) demonstration project [18]. The aim of this study is to report on the issues relating to patients with complex care needs and on the recommendations advanced during the summit in an effort to better integrate health, social, and community-based services for patients with chronic conditions and complex care needs.

## 2. Materials and Methods

A qualitative descriptive approach [19] was used to identify the issues raised and the recommendations advanced during the summit. Maximum variation sampling [20] (categories of participants and regions of Quebec) enabled us to gather 160 participants, including managers, researchers, specialized nurse clinicians and practitioners, decision makers, pharmacists, patient partners, social workers, doctors, community organization representatives, psychologists and a nutritionist, from IHSSCs and IUHSSCs, FMGs, and community-based organizations from 13 regions throughout Quebec (Table 1). Verbal informed consent was obtained from all participants at the beginning of the summit, after the research team presented the objectives of the event and the intention to disseminate the results. The Research Ethic Committee of the *Centre intégré universitaire de santé et de services sociaux de l’Estrie-CHUS* formally approved all procedures performed in this study (2014–2015) on 15 August 2014, including the verbal informed consent.

Mixed discussion groups of approximately ten participants, comprised of professionals and other stakeholders from different membership groups and backgrounds, focused on the main issues experienced in relation to patients with complex care needs around the question: “In your experience, what challenges related to interprofessional collaboration hinder patients with complex needs to have timely access to services meeting their needs?” Diversity within the groups aimed to promote exchanges that would help draw a comprehensive portrait of interprofessional and interorganizational collaboration issues. A plenary session helped identify the main issues. Following this, discussion groups comprised of approximately ten participants from the same professional field or membership group were tasked with collecting recommendations specific to their field of expertise. One of the participants in each discussion group was assigned to take notes. One member of the research team also took notes independently during the plenary sessions and group discussions. Other members of the research team acted as observers throughout the exchanges.

Following the summit, the research team and organizers of the day debriefed in order to highlight the themes that emerged from the discussions. The principal investigator and two research professionals then conducted a thematic analysis in which the data were reduced, organized and verified [21]. All notes and written reports of observations expressed as part of discussion groups, during plenary sessions as well as during the debriefing were coded by at least two authors using NVivo 11, and then grouped by theme, looking at convergences or contradictory observations [22]. Triangulation of sources (different categories of stakeholders) and researchers was used as a validation strategy [22,23].

## 3. Results

Seven main issues led to the formulation of seven recommendations that participants considered to be priorities.

### 3.1. Valuing the Experience of the Patient, Who Is the Expert on His/Her Complexity

Although patients manage their health on a day-to-day basis and are, as a result, experts on their own situation, their voice is not always heard. According to participating patients, the services trajectory should be integrated into the life trajectory: “The patient’s voice, experience, role, responsibility and life experiences must be recognized” “The patient is not just a disease”. Support from peer helpers for certain people with complex care needs should be promoted. Partnership with patients must be defined and promoted in order for them to contribute to support interprofessional collaboration and adapted organizational processes.

### 3.2. Early Detecting of a Non-Homogeneous Patient Population

Detection of patients with complex care needs means transcending stereotypes, because this population includes all age groups, with various conditions and is not homogeneous. Often passing “under the radar”, these patients are identified too late, once a critical event has occurred. Consequently, detection issues must be taken into account when developing strategies for early identification of these patients.

### 3.3. Defining Interprofessional Collaboration Based on Patient Needs

Overlapping of professional skills is a real issue in an interprofessional collaboration context. Although patients appreciate the contribution of every professional on the care team, interdisciplinarity often means repeating one’s story or receiving contradictory information. One professional said: “This is when repetition and contradiction occur; when we lose efficiency” However, working in an interdisciplinary setting also creates opportunities to work in a complementary way thanks to the proximity of health and social services professionals. Although the electronic file may be used as a platform to access and exchange information within the FMG, other more direct communication mechanisms must be introduced. The role of case managers can be supported from this perspective.

### 3.4. Conciliating a Registered Clientele-Based Logic with a Population-Based Logic

The mandates of FMGs, which serve a registered clientele, and those of the IHSSC/IUHSSC, which meet the needs of the population in a given territory, stem from two complementary logics. Tension between these two logics may create a conflict of allegiance for practitioners and put patients who are more vulnerable at risk if they are not registered with an FMG. This challenge requires that we “structure a collective action based on different paradigms”. Decision makers must also exercise motivational leadership to support teams during the transitions, devote the necessary time, support the development of practices, avoid duplication of services and implement a joint IHSSC/IUHSSC—FMG strategy to identify patients with complex care needs.

### 3.5. Working with the Community Sector

The casual nature of relationships between the health care network and community-based organizations combined with a mutual lack of awareness about their services mean that initiatives to bridge the gap remain uneven. However, these organizations are community resources that support people throughout their life trajectories and orient their actions in order to maintain independence. Playing a role as community “sensors”, they may constitute the only interface between the health care network and “orphan client groups”. The promotion of a collaborative approach must enable the creation and maintenance of partnerships and of more formal liaison mechanisms with community-based organizations.

### 3.6. Aligning SPOR Values with Existing Challenges to Primary Care Integration: The Importance of Intervention-Based Research

The presence of the Quebec SPOR SUPPORT Unit, of Réseau-1 Québec (Quebec Knowledge Network in Integrated Primary Health Care) and of two primary health care and social services university institutes in the province of Quebec generates a collective strength that can be highly impactful as well as being able to support transformations. Support from researchers may facilitate the use of evidence that can guide decision makers and professionals toward best practices. The following conditions should be put forward: development of outcome indicators, in collaboration with patients and their families; participation of decision makers, patient partners and health care professionals throughout the research process.

### 3.7. Promoting Resource Allocation that Is Consistent with Targeted Recommendations

It is important to support interprofessional collaboration by establishing priorities to this effect, by allocating the resources required to make it happen and by being creative with regard to the organization of services. It remains crucial to adequately fund community-based organizations so that they can serve their users and to invest the appropriate resources in the development and maintenance of partnerships so that the organizations have closer ties to the health and social services system.

## 4. Discussion

This summit held in Quebec and gathering 160 stakeholders highlighted seven recommendations to better integrate health, social, and community-based services for patients with chronic conditions and complex care needs. The raised issues and recommendations may be transferable to other Canadian provinces as well as to other industrialized countries. First, intervention coordination must recognize patients’ experience and their ability to play a role in their treatment plan [24]. Professionals must structure their intervention in accordance with the person’s life goals, their needs and their family’s needs [25]. Patients have a role to play in many different aspects, whether to help clarify the roles of professionals and organizations, to help develop a systematic process for consent to the exchange of information or to target priority research themes.

Next, although there exist various approaches to identifying patients with complex care needs internationally, many of the detection tools were developed for use in a hospital environment or for specific client groups (e.g., the elderly) [26]. Although the heterogeneity of this population complicates the use of a single detection tool, the generic approach of primary care [27] supports the use of a generic tool that is applicable to various forms of complexity. To date, the self-administered INTERMED questionnaire has proved valuable to assess the various aspects of complexity in a heterogeneous client group in a research context (medical, psychological, social and use of services) [28]. However, further study is required to assess its potential use in a clinical setting.

Furthermore, we see organizational structures of health care services intended for registered clients, as in Quebec’s FMGs, and population-based organizational structures for psychosocial services co-existing in the primary health care systems of the majority of Canadian provinces [29,30]. Participants in this consultation mentioned the possibility of conflicting allegiances for stakeholders who are likely to put certain vulnerable populations at risk. It remains crucial to properly develop the complementary aspects of these organizational structures.

Finally, in line with the SPOR vision, it is important to grow, support and sustain patient-oriented research that is collaborative, interdisciplinary, innovative and capable of enhancing health care for patients with complex care needs and improving clinical outcomes [18,31]. The participation of decision makers, patient partners and health care professionals as well as of stakeholders from various sectors is crucial throughout the research process.

There were also some limitations to this study. It would have been difficult to obtain a clear recording of discussions due to the numerous discussions taking place in the same room. Therefore, the analysis was conducted directly using the discussion notes and not a verbatim transcription of the discussions. However, triangulations of evaluators (number and diversity of perspectives) as well as the validation steps helped strengthen the trustworthiness of results. Furthermore, although data were only collected in Quebec, the majority of the issues that were raised and the formulated recommendations are transferable to other Canadian provinces and other industrialized countries with similar public health care systems.

## 5. Conclusions

The summit highlighted the importance of engaging all stakeholders in improvement of integrated care for patients with complex care needs. The resulting recommendations target shared priorities towards better health, social, and community-based services integration for these patients.

## Figures and Tables

**Table 1:** Profile of participants.

Profile of Participants from 13 Quebec Regions	
**Participants**	***n***
Community organization representatives	6
Decision makers	15
Doctors	9
Managers	52
Nutritionist	1
Patient partners	11
Pharmacists	13
Psychologists	2
Researchers	23
Social workers	10
Specialized nurse clinicians and practitioners	18
**Total number of participants**	**160**

## Data Availability

All data generated or analyzed during this study can be available if requested.
